# Insight into the Migration Routes of *Plutella xylostella* in China Using mt*COI* and ISSR Markers

**DOI:** 10.1371/journal.pone.0130905

**Published:** 2015-06-22

**Authors:** Jiaqiang Yang, Lixia Tian, Baoyun Xu, Wen Xie, Shaoli Wang, Youjun Zhang, Xiangjing Wang, Qingjun Wu

**Affiliations:** 1 Key Laboratory of Agriculture Biological Functional Gene of Heilongjiang Provincial Education Committee, Northeast Agricultural University, Harbin, China; 2 Institute of Vegetables and Flowers, Chinese Academy of Agricultural Sciences, Beijing, China; Institute of Zoology, CHINA

## Abstract

The larvae of the diamondback moth, *Plutella xylostella*, cause major economic losses to cruciferous crops, including cabbage, which is an important vegetable crop in China. In this study, we used the mitochondrial *COI* gene and 11 ISSR markers to characterize the genetic structure and seasonal migration routes of 23 *P*. *xylostella* populations in China. Both the mitochondrial and nuclear markers revealed high haplotype diversity and gene flow among the populations, although some degree of genetic isolation was evident between the populations of Hainan Island and other sampling sites. The dominant haplotypes, LX1 and LX2, differed significantly from all other haplotypes both in terms of the number of individuals with those haplotypes and their distributions. Haplotypes that were shared among populations revealed that *P*. *xylostella* migrates from the lower reaches of the Yangtze River to northern China and then to northeastern China. Our results also revealed another potential migration route for *P*. *xylostella*, i.e., from southwestern China to both northwestern and southern China.

## Introduction

The diamondback moth, *Plutella xylostella* (Lepidoptera: Plutellidae), which originated in the Mediterranean region or South Africa [1,2], is now considered a globally distributed insect pest [3]. *P*. *xylostella* can cause crop losses of more than 90% [4]. The total annual cost of damage caused by *P*. *xylostella* was recently estimated to be US$4–5 billions [5]. In China, *P*. *xylostella* has been a major pest of cruciferous crops since the 1970s and is now found in most cruciferous crop-growing areas. *P*. *xylostella* is considered a long-distance migratory species [3]. *P*. *xylostella* cannot survive in the cold winter conditions of regions such as western Canada, northern Japan, or northern China. However, re-colonization from warmer regions where populations persist year round occurs annually [6–9].

Many approaches have been used to determine the genetic structure of *P*. *xylostella*. Caprio and Tabashnik [10] in Hawaii and Kim [11] in South Korea used allozyme analyses and found little genetic differentiation among *P*. *xylostella* populations. Simple sequence repeat (SSR) markers have been used to determine the dispersal of *P*. *xylostella* populations in Australia where no population structure was observed [12]. Roux *et al*. [13] used ISSR markers to determine that each of 19 populations of *P*. *xylostella* from different areas of the world was genetically distinct. Another molecular marker that is widely used to study population structure is mitochondrial DNA (mtDNA) [14]. mtDNA is easy to extract, is maternally inherited, lacks genetic recombination, and has a relatively higher evolutionary rate compared with nuclear DNA [15,16]. Among several genes in the mtDNA, the cytochrome oxidase subunit I (mt*COI*) gene is highly variable at the DNA level [17]. Diversity as affected by genetic bottlenecks and migration events can be identified by testing the frequency of mt*COI* haplotypes [18,19]. In *P*. *xylostella*, the mt*COI* gene has 1531 bp [20] and greater variability and nucleotide diversity than other mitochondrial genes [21]. Previous studies have used the mt*COI* marker to characterize the genetic structure of *P*. *xylostella* in South Korea [22], China [23], Australia, and New Zealand [24]. Because of the short fragment lengths, small sample sizes, and narrow sampling ranges, however, these studies failed to accurately depict the migration route of *P*. *xylostella*. Hence, knowledge of *P*. *xylostella* migration routes and of geographical landscapes that act as barriers to dispersal remains unclear.

Because of their high variability, ISSR and mt*COI* markers are particularly suitable for evaluating the genetic structure and dispersal routes of insects [25–27]. In this study, we used 11 ISSR markers and one mt*COI* marker to determine the migration pattern and population genetic structure of *P*. *xylostella* sampled from 23 sites spread throughout China. We also compared the mt*COI* gene sequence data with published sequences from previous studies of *P*. *xylostella* in other regions.

## Materials and Methods

### Ethics Statement

The diamondback moth, *Plutella xylostella*, is the most significant insect pest of cruciferous plants. The study of this pest will provide information needed for its forecast and control. Sample collection did not involve endangered species. Thus, no specific permissions were required.

### Sampling sites

**Fig 1:**
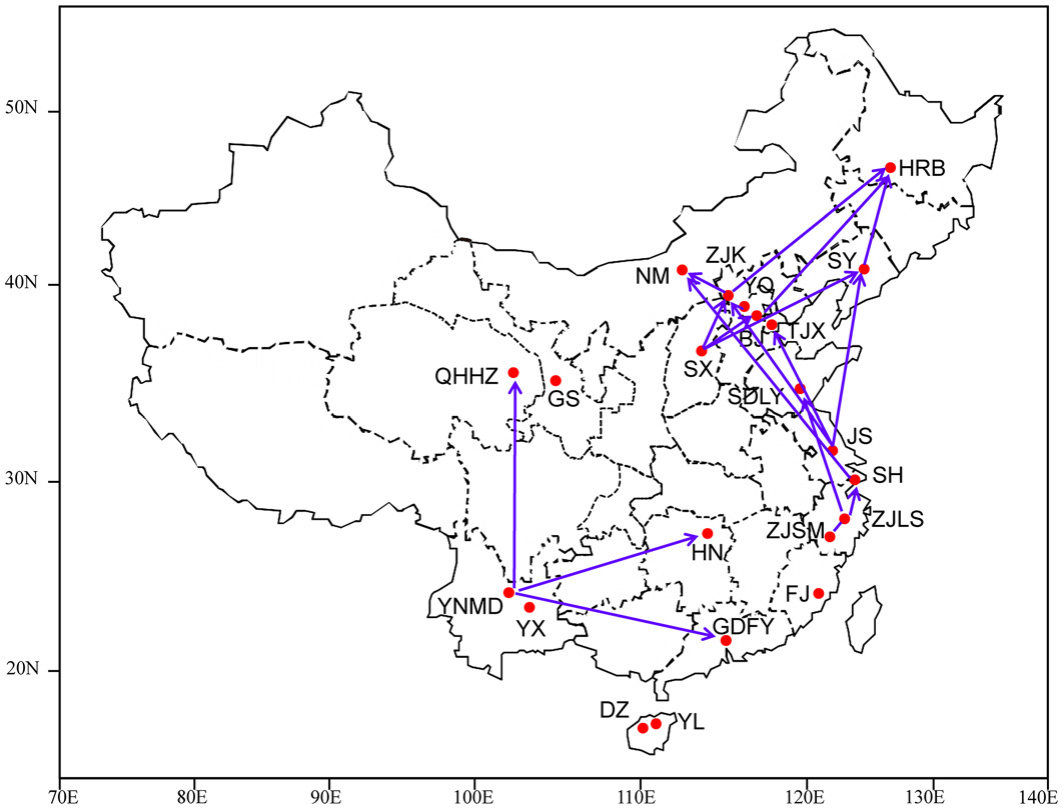
Locations where *Plutella xylostella* populations were sampled in China. Arrows indicate possible migration routes based on shared haplotypes. The software Adobe Photoshop CS6, Micosoft PowerPoint 2013 and Micosoft Word 2013 were used to create and modify this map.

We collected 620 *P*. *xylostella* individuals from 23 sampling sites (Fig 1). Adults were sampled using pheromone traps from May 2008 to October 2011 (Table 1). The collected materials were stored in 95% alcohol at -20°C.

**Table 1:** Details of *Plutella xylostella* populations collected in China.

Code	Location	Latitude	Longitude	Date of collection	No. of moths screened
HRB11	Haerbin, Heilongjiang	45.80N	126.53E	2011.5	33
HRB	Haerbin, Heilongjiang	45.80N	126.53E	2009.6	14
SY	Shenyang, Liaoning	41.80N	123.42E	2011.5	32
NM	Huhhot,Neimenggu	42.26N	118.88E	2011.6	31
ZJK	Zhangjiakou, Hebei	40.83 N	114.88E	2011.5	32
YQ	Yanqing, Beijing	40.48N	115.98E	2011.6	31
BJ	Haidian, Beijing	39.96N	116.27E	2011.5	36
TJX	Tianjin, Tianjin	39.36N	117.07E	2009.6	7
SX	Taiyuan,Shanxi	37.86N	112.43E	2011.10	28
SDLY	Laiyang, Shandong	36.99N	120.71E	2011.6	21
QHHZ	Huzhu, Qinghai	36.87N	101.96E	2011.6	33
GS	Lanzhou, Gansu	36.19N	103.78E	2008.7	8
JS	Yangzhou, Jiangsu	32.37N	119.39E	2011.8	31
SH	Shanghai, Shanghai	30.94N	121.49E	2011.4	35
ZJSM	Sanmen, Zhejiang	29.05N	121.47E	2011.5	36
ZJLS	Lishui, Zhejiang	28.38N	119.92E	2011.5	34
HN	Yueyang, Hunan	29.34N	113.12E	2011.5	22
FJ	Fuzhou, Fujian	26.12N	119.20E	2011.10	36
GDFY	Fanyu, Guangdong	22.95N	113.41E	2011.7	36
YX	Yuxi, Yunnan	24.35N	102.54E	2008.6	9
YNMD	Midu, Yunnan	25.34N	100.48E	2011.5	35
DZ	Danzhou, Hainan	19.55N	109.58E	2011.3	31
YL	Yunlong, Hainan	19.87N	110.47E	2010.5	9

### DNA extraction, PCR amplification, and sequencing

Whole moths were ground, and their DNA was extracted using the TIANamp genomic DNA kit and the manufacturer’s protocol (TIANGEN Biotech Co., Ltd., Beijing, China). A 704-bp fragment of the mt*COI* gene was amplified from all individuals analyzed using the primers and PCR procedures described by Li *et al*. [23]. After visual verification via gel electrophoresis, the PCR products were sequenced in both directions using an ABI 3730XL analyzer (Applied Biosystems, Foster City, CA).

### ISSR-PCR and electrophoresis

Inter-simple sequence repeat (ISSR) PCR amplification was performed using 11 primers (S1 Table) and the optimized procedures described in a previous study [28]. The reaction mixture (20 μL) contained: 1.5 mmol/L of Mg^2+^, 2.5 U of LA Taq DNA polymerase (Takara), 0.2 mmol/L of dNTPs mixture, 1.25 μmol/L of each primer, and 20 ng of DNA template. The cycling conditions were: initial denaturation of 5 min at 94°C; 35 cycles of 45 s at 94°C, 1 min at 40–58°C, and 1.5 min at 72°C; and a final extension of 10 min at 72°C, followed by storage at 4°C.

PCR products were subjected to electrophoresis on a 2% agarose gel using 1x Tris boric acid EDTA buffer at 150 V. The gels were visualized with ethidium bromide under UV.

### ISSR data analysis

ISSR DNA bands were scored qualitatively for the presence (1) or absence of bands (0) for each sample (each individual). This information was used to create matrices of the 11 primers. The software POPGENE 1.32 [29] was used to calculate the percentage of polymorphism (Ppl), Shannon’s information index (I), Nei’s gene diversity (He), the effective number of alleles (Ne), the total genetic diversity for species (Ht), the genetic diversity within the population (Hs), the coefficient of gene differentiation (Gst), and the gene flow among populations (Nm). The dendrogram of 23 *P*. *xylostella* populations was constructed by cluster analysis using the unweighted pair group method of the arithmetic averages (UPGMA) in the software POPGENE 1.32.

### Population genetics and phylogenetic analysis

To assess how genetic diversity differed among geographic populations, we calculated haplotype diversity (Hd), nucleotide diversity (Pi), and the mean number of pair-wise differences using DnaSP5 [30]. Arlequin 3.5 was used to estimate genetic differentiation between populations (F_ST_) [31]. To determine the relationship among *P*. *xylostella* haplotypes, a parsimony haplotype network with 95% statistical support was obtained using TCS 1.21 [32]. The statistical parsimony haplotype networks are reported here to provide representations of gene genealogies at the population level [33].

To compare the relationship of the haplotypes between China and neighboring country, the available *mtCOI* gene sequences of *P*. *xylostella* were downloaded from GenBank. A total of 296 sequences with 658-bp homologous regions were used for further analysis (GenBank accession numbers KC154937–KC155152 and DQ076332– DQ076411). Thirty of 296 sequences were sampled from South Korea, and the remaining sequences were sampled from China. With the program MEGA 4.0 and the K2P model [34], a neighbor-joining (NJ) tree was created to provide a graphic representation of haplotype divergence.

A Mantel test for isolation-by-distance, as revealed by a correlation between Fst values and log-geographic distances, was performed using IBDWS 3.23 [35]. We used a reduced major axis (RMA) regression analysis to estimate the slope and intercept of the isolation-by-distance relationship, and calculated from 10000 randomizations.

### Demographic analysis

To detect evidence of range expansion in the populations of *P*. *xylostella*, Tajima’s D [36] and Fu’s F_S_ [37] tests were performed. A significant Tajima’s D value could result from factors other than selective effects, such as population expansions, bottlenecks, or heterogeneity of mutation rates [38]. Similarly, Fu’s F_S_ test is very sensitive to demographic population expansion, which generally leads to larger negative values [39]. Both Tajima’s D and Fu’s F_S_ tests were calculated by Arlequin 3.5.

## Results

### General features of mtCOI gene sequences

Alignment of a 704-bp fragment of the *P*. *xylostella* mt*COI* gene from 620 individuals revealed 193 haplotypes (GenBank accession numbers KM588399–KM588591) among the 23 populations (Table 1 and Fig 1). These haplotypes contained 99 polymorphic sites (14.06% variation), consisting of 58 parsimony-informative sites and 41 singleton-variable sites. Nucleotide diversity among the 23 populations ranged from 0.212 to 0.529 (Table 2).

**Table 2:** Results of genetic diversity and neutrality tests based on *mtCOI* sequences for populations of *Plutella xylostella* in China.

Population	H	Hd	Pi(%)	k	Tajima’s D	Fu’s F_S_
HRB11	25	0.968±0.020	0.461±0.910	3.242	-1.73411*	-24.161
HRB	11	0.956±0.045	0.379±0.581	2.670	-1.39345	-7.00444
SY	17	0.891±0.040	0.344±0.600	2.423	-1.43889	-11.2692
NM	16	0.912±0.033	0.298±0.462	2.099	-1.16430	-11.1953
ZJK	18	0.911±0.035	0.320±0.741	2.252	-1.96549**	-13.9527
YQ	19	0.908±0.042	0.320±0.640	2.254	-1.71046*	-16.4052
BJ	14	0.816±0.054	0.212±0.514	1.495	-1.90664**	-9.81075
TJX	6	0.952±0.096	0.473±0.522	3.333	-0.49212	-1.99294
SX	18	0.897±0.051	0.395±0.657	2.783	-1.39099	-13.1206
SDLY	9	0.824±0.060	0.241±0.434	1.695	-1.87758*	-3.87167
QHHZ	18	0.896±0.043	0.307±0.490	2.163	-1.22098	-14.0939
GS	5	0.786±0.151	0.380±0.548	2.679	-1.51227	-0.52342
JS	16	0.883±0.045	0.291±0.423	2.046	-1.00830	-11.1824
SH	18	0.921±0.029	0.344±0.690	2.424	-1.69613*	-12.2197
ZJSM	17	0.921±0.027	0.335±0.617	2.357	-1.52067*	-10.5413
ZJLS	22	0.947±0.026	0.393±0.764	2.766	-1.67027*	-18.939
HN	12	0.909±0.044	0.359±0.468	2.528	-0.81289	-5.63523
FJ	12	0.903±0.023	0.299±0.514	2.102	-1.36184	-4.47463
GDFY	22	0.930±0.029	0.411±0.822	2.897	-1.71472*	-17.3971
YX	8	0.972±0.064	0.458±0.679	3.222	-1.55699	-4.0932
YNMD	22	0.938±0.029	0.365±0.793	2.571	-1.85376*	-19.5432
DZ	8	0.718±0.080	0.324±0.391	2.284	-0.54500	-0.77414
YL	8	0.972±0.064	0.529±0.679	3.722	-1.05826	-3.61144

H, number of haplotypes; Hd, haplotype diversity; Pi, nucleotide diversity; k, average number of nucleotide differences.

*P<0.05

**P<0.01.

### Population genetic structure

**Fig 2:**
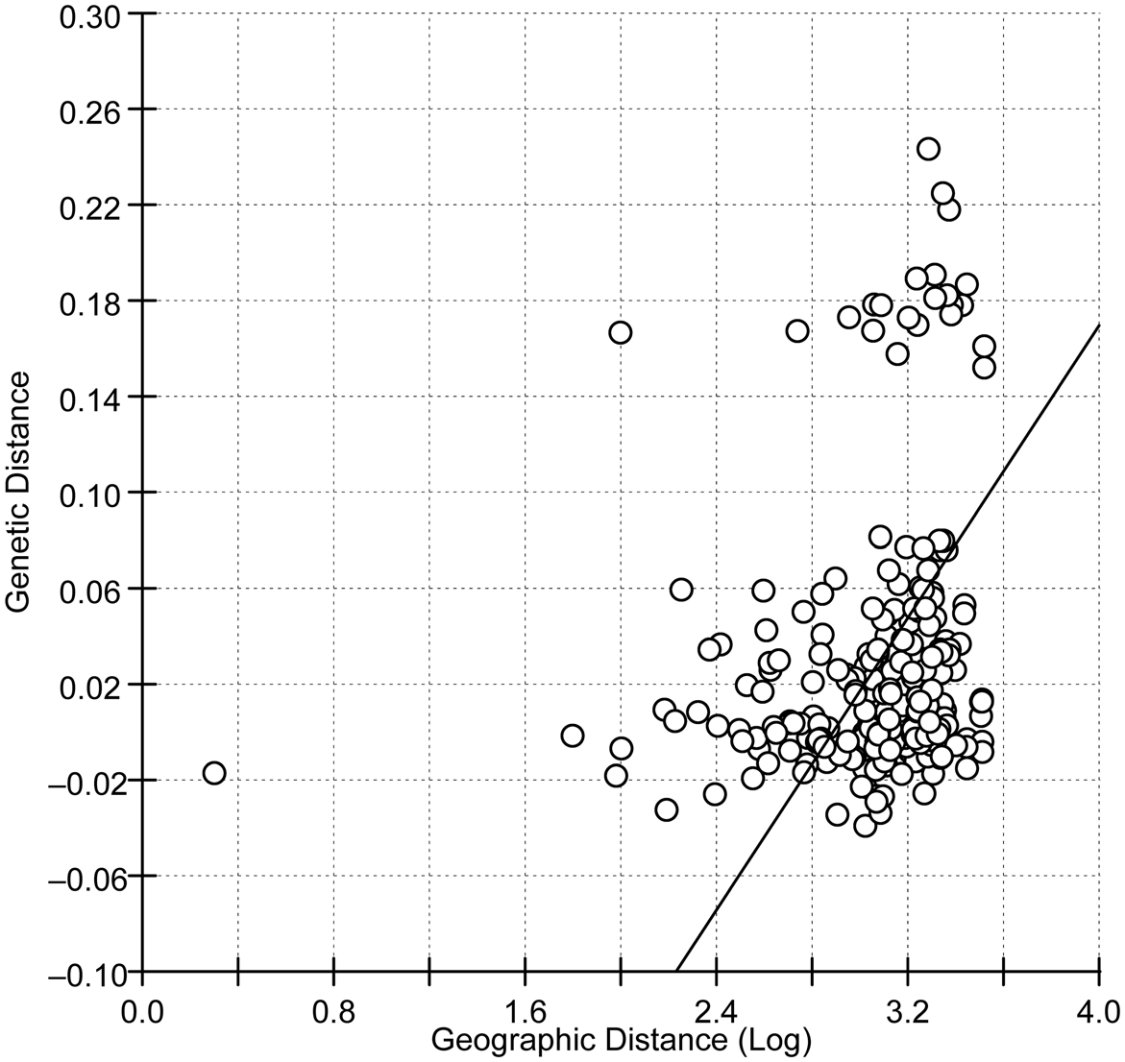
Results of a Mantel test showing the correlation between pairwise Fst values and the logarithm of geographic distances of Chinese populations of *Plutella xylostella*. Reduced major axis (RMA) analyses were calculated from 10000 randomizations.

The F_*ST*_ values among the 23 populations ranged from -0.03916 to 0.2433 (S2 Table). Neutrality tests showed that the Tajima’s D and Fu’s F_*S*_ values of all *P*. *xylostella* populations were negative (Table 2), which does not support a neutral model of evolution and suggests that population expansion, genetic hitchhiking, and/or selection have occurred during the evolutionary history of *P*. *xylostella*. The Mantel test revealed a weak but significant positive correlation between genetic distance and the log of geographical distance (r = 0.1885, *P* = 0.0030; Fig 2).

### Haplotype distribution and network

Of the 193 haplotypes, 38 were shared among all the populations (Table 3). The other 155 haplotypes were unique, with each sampled population containing unique haplotypes, suggesting some degree of isolation between the populations (Table 3). The haplotype LX1 (n = 123) was the most common haplotype and was shared by all 23 populations. The haplotype LX2 (n = 108) differed by only one nucleotide from LX1 and was the next most widely distributed haplotype and was detected in all populations except the two on Hainan Island (DZ and YL; Table 3). Twenty of the 38 shared haplotypes were observed in only two individuals sampled from two populations (Table 3).

**Table 3:** The number of shared haplotypes for each of the sampled populations of *Plutella xylostella* in China.

Population	Shared haplotypes																																					
	LX1	LX2	LX4	LX11	LX12	LX15	LX24	LX25	LX26	LX30	LX35	LX36	LX37	LX38	LX39	LX41	LX42	LX44	LX45	LX51	LX53	LX56	LX63	LX69	LX88	LX92	LX95	LX97	LX104	LX109	LX112	LX115	LX122	LX139	LX144	LX150	LX152	LX157
HRB11	5*	4				1	1				2							1			1							1										
HRB	3	2								1	1	1	1																									
SY	6	9			1		1		1	1			1					3														1	1					
NM	6	7			1	2	1				2				1		1	1							1				3									
ZJK	8	5							1		1			1				4							1	1	1	1										
YQ	9	4				1		1					1			1	1	1																				
BJ	14	7						1				2	1	1							1									3	1							
TJX	2	1																		1																		
SX	3	9				1			2					1	1												1				1		1					
SDLY	6	7						1										2				1																
QHHZ	4	10			3	1					1		2	1	1		1	1					1											1				
GS	4	1																																				
JS	10	5		1	1	2		1	2											1						1				1		1						
SH	8	5			2		1				2						1	4											2						1	1	1	
ZJSM	4	8	1	1	1		3				5		1									1	1												1		2	2
ZJLS	7	4	1		1	1	2				1		1	2																						1		1
HN	6	3												1	1	1	1	2	1																			
FJ	5	2											4	1	7																							
GDFY	7	7						1	1				1	1	2			1					1	2														
YX	1					2																																
YNMD	4	8						1			2			1			1	2	1					2						1				1				
DZ	1				2							2	2			4																						
YL	1			1	1																																	

*: The number in the table represents the number of individuals with a particular haplotype in that population.

**Fig 3:**
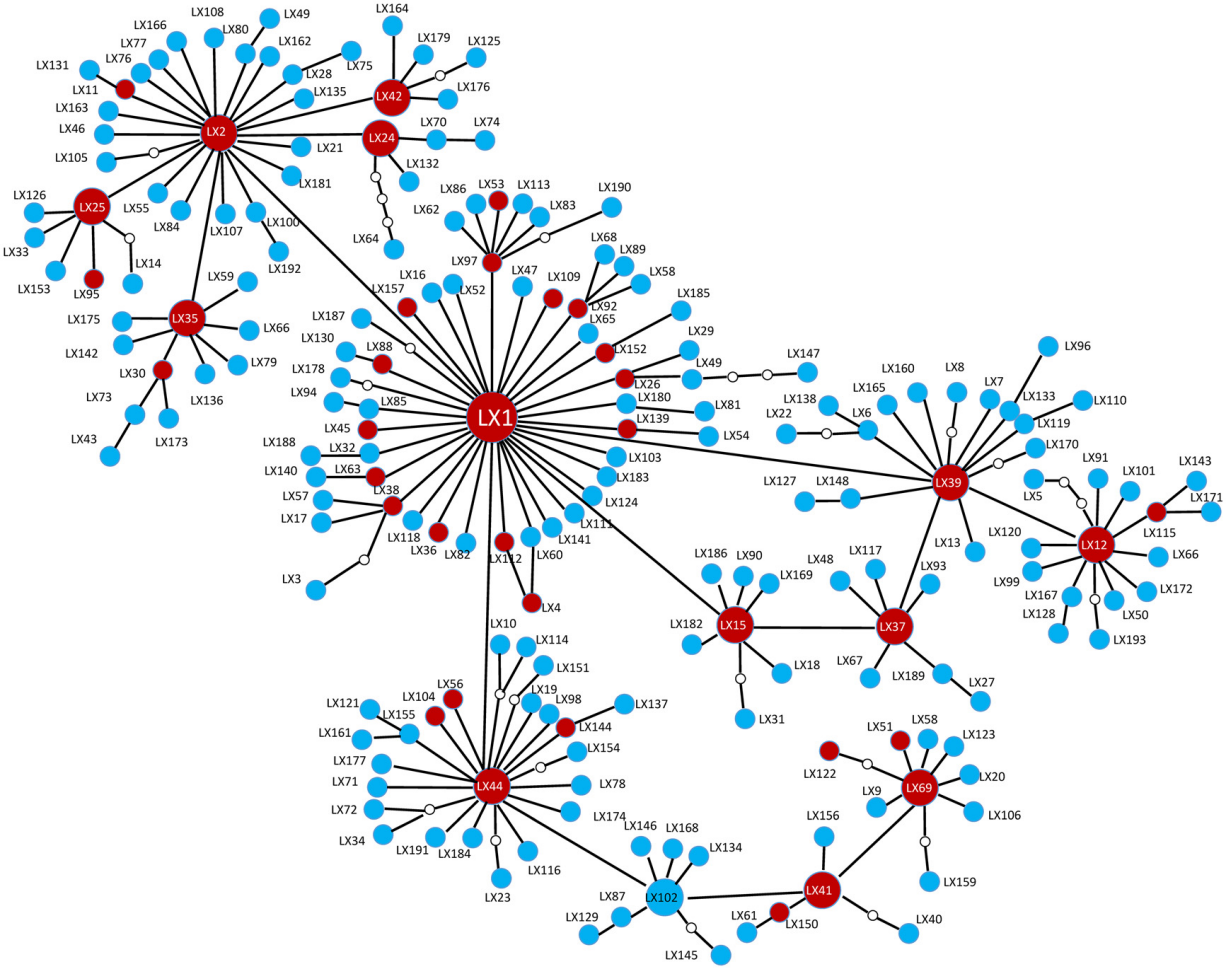
Statistical parsimony network of *Plutella xylostella* mt*COI* haplotypes. The red and blue circles represents shared and unique haplotypes, respectively. Haplotype names are beside the circles. The small circles indicate the presence of missing intermediates, while the connections are based on the set of plausible solutions with a 95% of parsimony probability.

**Fig 4:**
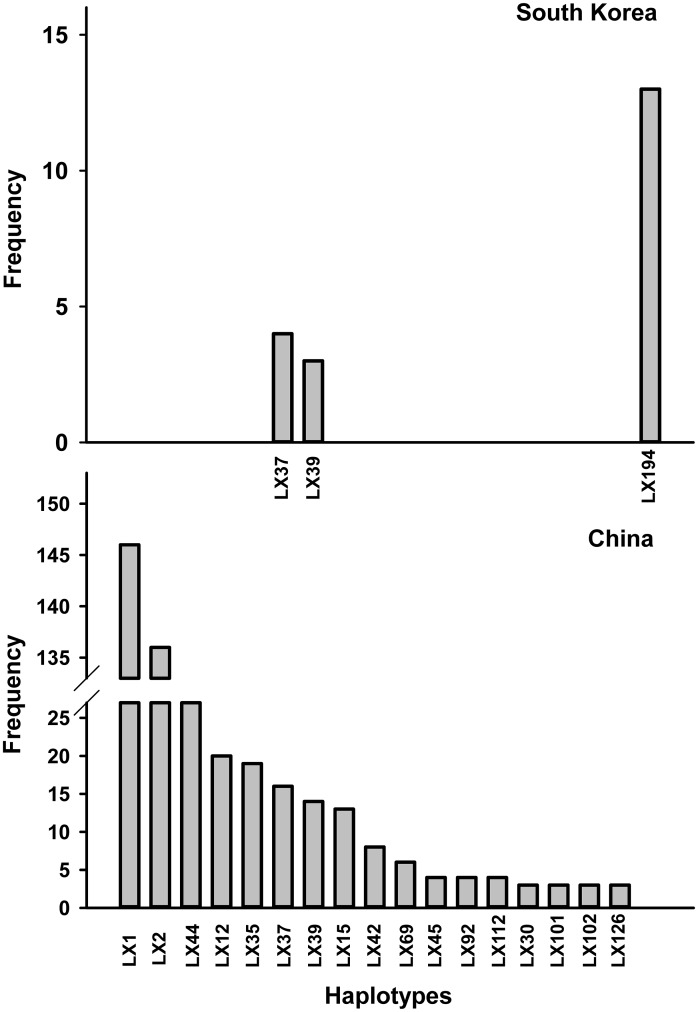
Comparison of *mtCO1* haplotype frequency distributions in samples of *Plutella xylostella* from South Korean (top) and in *Plutella xylostella* populations across China (bottom). Haplotypes with frequency ≤ 2 are not shown.

A statistical parsimony network analysis of the relationships among mt*COI* haplotypes revealed that haplotype LX1 occupied a central position in all populations. Unique haplotypes were located at the edges of the network (Fig 3). When we compared the sequences of the *mtCOI* gene with 658-bp homologous regions from GenBank (GenBank accession numbers KC154937–KC155152 and DQ076332–DQ076411), the number of haplotype increased to 324. Two South Korean haplotypes were identical to haplotypes LX37 and LX39 from China, but the most common haplotype in South Korea (LX194) was absent in China. In addition, neither haplotype LX1 nor LX2 was found in South Korea (Fig 4). A neighbor-joining tree constructed for the 324 haplotypes revealed that most haplotypes clustered as a single clade (S1 Fig).

### ISSR profile and analysis

Using 11 ISSR primers (S1 Table), we repeatability detected a total of 230 loci (bands) from the 23 populations. The percentage of polymorphism (Ppl) ranged from 62.17 to 86.96 (S3 Table). At the species level, all individuals possessed a unique ISSR genotype, suggesting a high degree of genetic variation within populations.

The percentage of polymorphism (Ppl), Shannon’s information index (I), Nei’s gene diversity (He), and effective number of alleles (Ne) within population are summarized in S3 Table. The population ‘DZ’ located on Hainan Island showed the lowest effective number of alleles (1.1233 ± 0.1864), lowest Nei’s gene diversity (0.0911 ± 0.1151), and lowest Shannon’s information index (0.1625 ± 0.1781). Another Hainan Island population, YL, also exhibited less diversity than the other populations (S3 Table). G_*st*_ was estimated as 0.0858, indicating that 8.58% of the genetic variability was distributed among populations. The number of genetic migrants (Nm) was 5.3277, which suggests a high level of gene flow among the sampled populations (S4 Table).

**Fig 5:**
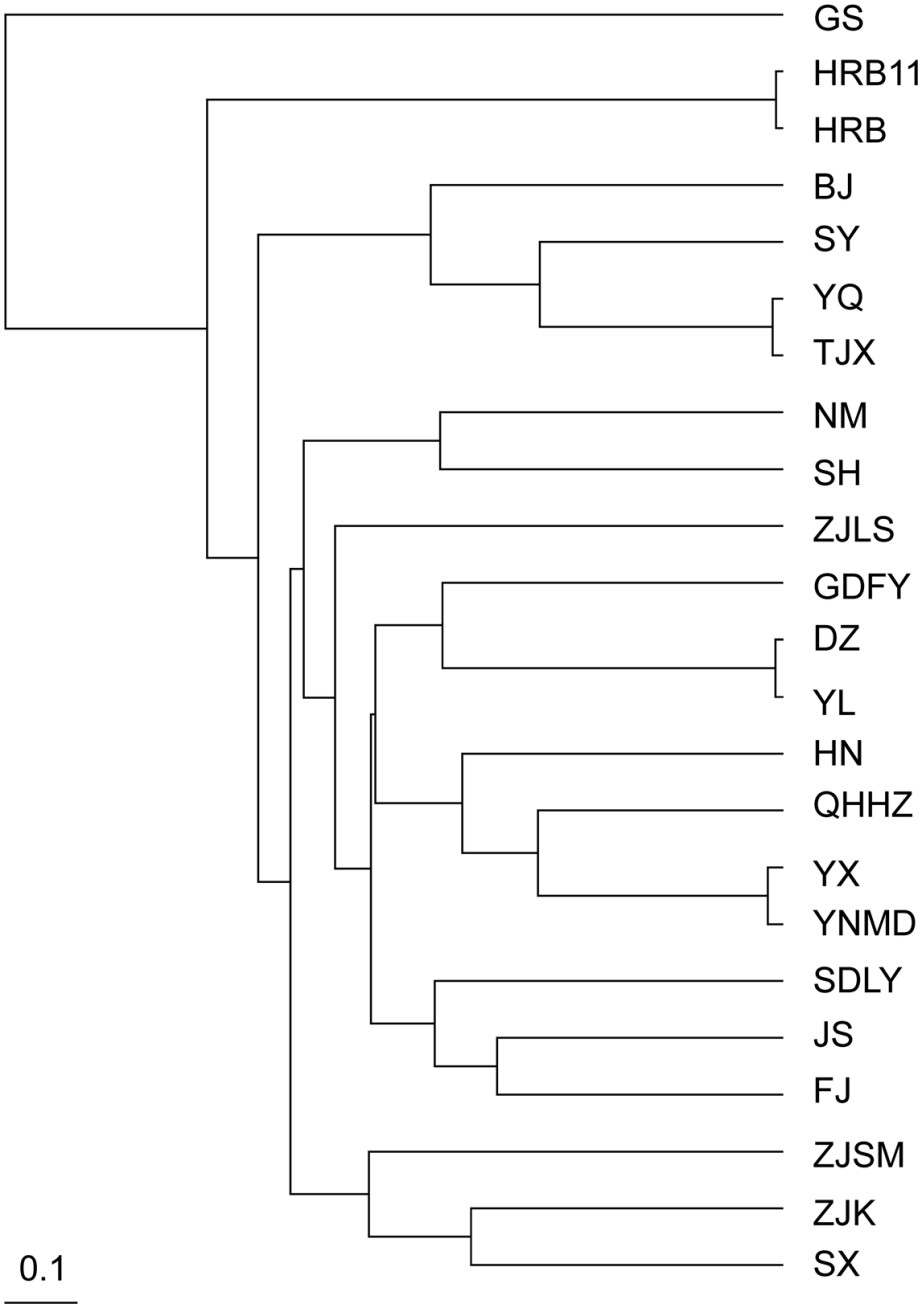
UPGMA dendrogram of 23 populations of *Plutella xylostella* in China based on ISSR markers.

We constructed a dendrogram using UPGMA analysis to infer phylogenetic relationships among the *P*. *xylostella* populations. The populations BJ, SY, YQ, and TJX in seasonally suitable areas clustered together (Fig 5). The clustering roughly correspond to geographical distance.

## Discussion

By analyzing mt*COI* and ISSR data, we have determined that *P*. *xylostella* populations in China exhibit high genetic variability at the nucleotide level and high mitochondrial haplotype diversity. The haplotype distribution was characterized by a large number of unique haplotypes isolated in each population’s home range; only a few haplotypes were widely shared among the 23 populations. While only three haplotypes were reported in Australian populations of *P*. *xylostella* based on a 257-bp fragment of the mt*COI* gene [24], we found 193 haplotypes. In our demographic analysis, the values of Tajima’s D and Fu’s F_S_ tests were negative, suggesting that the haplotype diversity was due to population expansion and genetic selection. Chemical compounds and insecticides with high mutagenicity increase the number of mutations in resistant individuals. Many studies have shown that *P*. *xylostella* has developed resistance to a variety of insecticides in the field [40–47]. Different levels of resistance have even been reported in two geographically close populations [10,40,41,48]. The bottleneck effect caused by excessive use of insecticides selects for differing haplotypes and leads to divergence between populations. Differences in local survival rates and insecticide-induced selection pressure might be the major force leading to high haplotype diversity [21,22]. Another factor that might explain the high haplotype diversity could be the widespread and continuous planting of cruciferous crops grown in China, which provide a steady supply of highly suitable host plants for *P*. *xylostella*. Host plant shift is generally thought to be an important cause of genetic polymorphism in host plant adaptation [49]. Genetic analyses of *P*. *xylostella* populations have shown that this insect has substantial genetic plasticity in that alleles enabling survival on different hosts [50]. You *et al*. [51] also reported that *P*. *xylostella* coevolved with its host plant.

Of the 38 shared haplotypes found in our study, LX1 and LX2 were found in almost all of the *P*. *xylostella* populations, indicating their significance. Although both of these haplotypes were widely distributed, LX2 was not found in the populations located on Hainan Island (DZ and YL). Based on these finding, we infer that *P*. *xylostella* populations in China originated from one female founder moth with haplotype LX1.

*P*. *xylostella* has historically been considered to be capable of long-distance migration [3]. More recent studies, however, suggest that the dispersal ability of this species is limited [52,53]. A mark-recapture study showed that adult males and females of *P*. *xylostella* had similar dispersal abilities and that fewer than 1% moved more than 200 m from their release point within 5 to 9 days [52]. In Hawaii, genetically distinct populations of *P*. *xylostella* are separated by less than 10 km [53]. Our results showed that the majority of haplotypes (155 of 193) were unique, indicating that most haplotypes are locally restricted. In addition, the Mantel test revealed a positive correlation between genetic distance and geographic distance. The UPGMA dendrogram also revealed that the populations closely located geographically clustered in the same clades. We found that seven haplotypes were shared by populations SH and ZJSM, of which two haplotypes (LX144 and LX152) were found only in these two close locations (a distance of ~200 km). Similarly, five haplotypes were shared by populations NM and ZJK in northern China, with only a single individual possessing haplotype LX88 in each population. The number of shared haplotypes was reduced as distance increased. The limited number of shared haplotypes and the high level of gene flow that we observed among populations indicates that *P*. *xylostella* can migrate only short distances. Short distance migration enables *P*. *xylostella* to move in a step-by-step manner in search of seasonally suitable areas. Annually, *P*. *xylostella* is able to migrate from regions where it can overwinter to regions where it cannot overwinter because the winter temperatures are too low [3,6–9,21,54].

Several shared haplotypes were distributed in different regions of China, and this information reveals potential relationships between populations. *Plutella xylostella* persists year-round in subtropical and tropical regions. The widely distributed shared haplotypes suggest the gene flow in those region. Furthermore, in the temperate zone, where the JS and ZJK populations are located, shared haplotypes become more common because of migration. Besides the widely distribution haplotypes, the point-to-point haplotypes (haplotypes shared by only two populations) revealed the migration direction between *P*. *xylostella* populations. Three point-to-point haplotypes of the JS population were shared with other temperate zone populations located in northern China (TJX, ZJK, and SY). Similarly, the ZJK population also shared point-to-point haplotypes with SX, NM, and HRB populations. From these results, we infer that one possible migration of *P*. *xylostella* begins in southeastern China (JS, ZJLS, and ZJSM populations) and progresses north (ZJK and BJ populations) into northeastern China (SY and HRB populations). In addition, the YNMD population in southwestern China shared point-to-point haplotypes with QHHZ, HN, and GDFY populations, which suggests another migration route that begins near the location of the YNMD population. The YX, YNMD, QHHZ, and HN populations clustered together in the UPGMA dendrogram, which is highly consistent with the hypothesis stated above. Recent studies have shown that the Qinling Mountain is a barrier for *P*. *xylostella* migration and gene flow [55]. Interestingly, we found that none of the point-to-point haplotypes appeared to cross this mountain range.

The factors affecting dispersal and gene flow of *P*. *xylostella* are complex. Wind-enabled flight enables these insects to fly further than would be possible by powered flight alone [56,57]. In China, the East Asian subtropical monsoon provides *P*. *xylostella* with an annual opportunity to re-colonize from southern to northern regions [21]. In southwestern China, the Bengal Bay monsoon begins in May, concurrent with the *P*. *xylostella* outbreak in Yunnan Province. This indicates that *P*. *xylostella* may use the strong southwesterly winds caused by the monsoon to migrate into northwestern and southern China. Alternatively, however, the distribution of *P*. *xylostella* might simply reflect the extended cultivation of its host plants [58]. Indeed, the long-distance transport of cabbage plants probably has had a greater effect on genetic exchange of *P*. *xylostella* than natural gene flow [13].

In this study, the two most common and widely distributed haplotypes, LX1 and LX2, were absent from Hainan Island, and no point-to-point haplotype was found between the populations on Hainan Island and other sites. These findings indicate that the ocean is an effective barrier for *P*. *xylostella* dispersal. This is supported by the results of the ISSR data, which showed that the Hainan populations were genetically distinct from the other populations. Further evidence that oceans can act as barriers for *P*. *xylostella* dispersal was that both LX1 and LX2 were absent from South Korea and that the most common haplotype of South Korea (LX194) was not found in China. This indicates that *P*. *xylostella* populations in China and South Korea are isolated by the barrier of the East China Sea. A former study of *P*. *xylostella* populations suggested that there were barriers to gene flow and some degree of genetic isolation between Australia and New Zealand [24].

In conclusion, we used both mitochondrial and nuclear markers to analyze the genetic structure of *P*. *xylostella* populations in China and found high haplotype diversity and high gene flow among geographic populations. Nevertheless, the high pairwise F_ST_ values between Hainan Island and mainland China populations indicate strong genetic differentiation when dispersal is limited by oceanic barriers. The distribution of shared haplotypes among our sampled populations reveals two primary migration routes for *P*. *xylostella* in China; the first route begins in southeastern China (in the lower reaches of the Yangtze River) and progresses through northern and northeastern China, and the second route begins in southwestern China and progresses through northwestern and southern China.

## Supporting Information

S1 Fig NJ tree of 324 mtCOI haplotypes of *Plutella xylostella* in China and South Korea. (PDF)

S1 Table ISSR-PCR primers and their annealing temperatures. (PDF)

S2 Table Pair-wise F_ST_ values between sampled populations of *Plutella xylostella* in China. (PDF)

S3 Table Genetic variability of the *Plutella xylostella* based on ISSR data. (PDF)

S4 Table Estimated gene diversity for 23 *Plutella xylostella* populations as indicated by 11 ISSR makers. (PDF)
