# Supplementary material for: Insight into the Migration Routes of Plutella xylostella in China Using mtCOI and ISSR Markers
Source: PLoS One. 2015 Jun 22;10(6):e0130905. doi: 10.1371/journal.pone.0130905 (PMC4476569; doi:10.1371/journal.pone.0130905)
Supplement: S1 Table — (PDF) [file pone.0130905.s002.pdf]

S1 Table ISSR-PCR primers and their annealing temperatures.

Primer code	Primer sequence (5'-3')	Primer abbreviation	Annealing T℃
807	AGAGAGAGAGAGAGAGT	$(AG)_8T$	52
823	TCTCTCTCTCTCTCTCC	$(TC)_8C$	54
826	ACACACACACACACACC	$(AC)_8C$	53
840	GAGAGAGAGAGAGAGAYT	$(GA)_8YT$	58
873	GACAGACAGACAGACA	$(GACA)_4$	44
879	CTTCACTTCACTTCA	$(CTTCA)_3$	40
886	VDVCTCTCTCTCTCTCT	$VDV(CT)_7$	41
887	DVDTCTCTCTCTCTCTC	$DVD(TC)_7$	40
888	BDBCACACACACACACA	$BDB(CA)_7$	50
903	RYCACACACACACACA	$RY(CA)_7$	55
906	WBGACAGACAGACAGACA	$WB(GACA)_4$	58

With B = (T, C, or G); D = (A, T, or G); R = (A or G); W = (A or T); Y = (C or T); V = (A, C, or G).
