# Supplementary material for: Insight into the Migration Routes of Plutella xylostella in China Using mtCOI and ISSR Markers
Source: PLoS One. 2015 Jun 22;10(6):e0130905. doi: 10.1371/journal.pone.0130905 (PMC4476569; doi:10.1371/journal.pone.0130905)
Supplement: S2 Table — (PDF) [file pone.0130905.s003.pdf]

S2 Table Pair-wise F_{ST} values between sampled populations of *Plutella xylostella* in China.

Population	F_{ST} value											
	HRB11	HRB	SY	NM	ZJK	YQ	BJ	TJX	SX	SDLY	QHHZ	GS
HRB11												
HRB	-0.01717											
SY	0.00451	-0.00783										
NM	-0.00574	-0.01247	-0.00705									
ZJK	-0.00322	-0.0119	-0.00527	-0.00251								
YQ	-0.0003	-0.01515	0.00654	0.00091	-0.00685							
BJ	**0.02739**	0.00012	0.02089	0.0196	0.00934	-0.00149						
TJX	-0.02287	-0.03916	-0.01349	-0.01926	-0.02596	-0.03248	-0.01824					
SX	0.01185	0.00626	-0.00754	-0.00125	0.0169	**0.02598**	**0.04255**	0.00213				
SDLY	0.0176	0.00325	-0.01679	-0.00246	-0.00423	0.00354	0.00364	-0.01304	0.00171			
QHHZ	0.00903	-0.00047	-0.01019	-0.0061	0.01431	0.0208	**0.04008**	0.00137	-0.01124	0.00117		
GS	0.0245	-0.00075	0.03069	0.02227	0.00104	-0.01575	-0.02665	-0.03374	**0.06412**	0.01116	0.05947	
JS	0.0068	-0.00818	0.00723	0.00196	-0.00296	-0.01038	-0.00562	-0.0345	0.02405	0.00372	0.02262	-0.01747
SH	-0.00622	-0.01221	-0.00281	-0.0105	-0.01293	-0.00405	0.01692	-0.02273	0.02212	0.0032	0.01014	0.00863
ZJSM	0.0003	-0.00542	0.007	-0.00052	**0.01841**	**0.02563**	**0.04881**	0.00202	0.00854	0.02172	0.00183	**0.05946**
ZJLS	-0.00744	-0.01737	0.00622	-0.00246	-0.00167	-0.00426	0.01514	-0.02682	**0.01612**	0.01709	0.01092	0.00902
HN	-0.00094	-0.01075	0.00223	0.00009	-0.0139	-0.01219	0.00528	-0.02907	0.02254	0.00163	0.01785	-0.00676
FJ	**0.03792**	0.02749	**0.06039**	**0.04908**	**0.05077**	**0.04594**	**0.0771**	0.02933	**0.06179**	**0.08143**	**0.05598**	**0.07662**
GDFY	-0.00297	-0.01511	-0.00684	-0.00574	-0.00541	-0.00341	0.01272	-0.02559	-0.00101	-0.0027	-0.00156	0.01452
YX	0.00677	0.01272	**0.05301**	0.02589	0.03445	0.02469	0.07591	0.00579	0.05044	**0.0798**	**0.05097**	0.06752
YNMD	-0.00407	-0.00829	-0.00616	-0.00549	0.00141	0.01168	**0.03349**	-0.01026	-0.00459	0.00271	-0.00663	0.04704
DZ	**0.15199**	**0.16086**	**0.18676**	**0.1781**	**0.17822**	**0.17421**	**0.21792**	**0.18223**	**0.1908**	**0.22482**	**0.18107**	**0.24333**
YL	0.01358	0.01272	0.04957	0.03681	0.03445	0.03203	**0.07591**	0.00579	**0.05821**	**0.0798**	**0.04763**	0.06752

Significant pairwise ($P < 0.05$) differences are indicated in bold.

S2 Table Continued

Population	F_{ST} value										
	JS	SH	ZJSM	ZJLS	HN	FJ	GDFY	YX	YNMD	DZ	YL
HRB11											
HRB											
SY											
NM											
ZJK											
YQ											
BJ											
TJX											
SX											
SDLY											
QHHZ											
GS											
JS											
SH	0.00262										
ZJSM	**0.02893**	0.00835									
ZJLS	-0.00036	-0.00377	0.00464								
HN	-0.00272	-0.00954	**0.02597**	-0.00321							
FJ	**0.05774**	**0.05023**	**0.05905**	**0.03664**	**0.04065**						
GDFY	-0.00049	-0.00106	0.00883	-0.00395	-0.00619	**0.03257**					
YX	0.02586	0.03138	**0.0446**	0.01275	0.03256	0.05158	0.02999				
YNMD	**0.01753**	0.00129	-0.0007	0.00428	0.00523	**0.05168**	-0.00755	0.03449			
DZ	**0.1892**	**0.16978**	**0.17289**	**0.15767**	**0.17832**	**0.17809**	**0.1673**	**0.173**	**0.16739**		
YL	0.0367	0.02494	**0.0385**	0.01609	0.03256	**0.05158**	0.02999	0.01563	0.03449	**0.1666**	

Significant pairwise (*P* < 0.05) differences are indicated in bold.
