# Supplementary material for: Insight into the Migration Routes of Plutella xylostella in China Using mtCOI and ISSR Markers
Source: PLoS One. 2015 Jun 22;10(6):e0130905. doi: 10.1371/journal.pone.0130905 (PMC4476569; doi:10.1371/journal.pone.0130905)
Supplement: S3 Table — (PDF) [file pone.0130905.s004.pdf]

S3 Table Genetic variability of the *Plutella xylostella* based on ISSR data.

Population	Ne	He	I	Npl	Ppl
HRB11	1.2632±0.3175	0.1673±0.1665	0.2725±0.2305	185	80.43
HRB	1.2750±0.3128	0.1767±0.1623	0.2892±0.2220	196	85.22
SY	1.1672±0.2085	0.1213±0.1254	0.2123±0.1873	172	74.78
NM	1.2358±0.2645	0.1606±0.1445	0.2698±0.2041	190	82.61
ZJK	1.2220±0.2636	0.1506±0.1470	0.2525±0.2104	180	78.26
YQ	1.2007±0.2451	0.1394±0.1390	0.2376±0.2016	179	77.83
BJ	1.1810±0.2439	0.1247±0.1415	0.2106±0.2092	153	66.52
TJX	1.2180±0.2428	0.1526±0.1356	0.2607±0.1934	194	84.35
SX	1.2402±0.2810	0.1593±0.1552	0.2626±0.2208	178	77.39
SDLY	1.1887±0.2509	0.1296±0.1419	0.2194±0.2076	162	70.43
QHHZ	1.2574±0.2765	0.1717±0.1534	0.2830±0.2162	192	83.48
GS	1.2735±0.3023	0.1772±0.1619	0.2873±0.2278	181	78.70
JS	1.2577±0.2803	0.1717±0.1522	0.2842±0.2128	194	84.35
SH	1.2310±0.2442	0.1606±0.1395	0.2698±0.2022	184	80.00
ZJSM	1.2358±0.2767	0.1571±0.1540	0.2597±0.2197	180	78.26
ZJLS	1.2498±0.2805	0.1655±0.1565	0.2709±0.2237	178	77.39
HN	1.2429±0.2754	0.1626±0.1517	0.2691±0.2161	182	79.13
FJ	1.2183±0.2605	0.1486±0.1463	0.2499±0.2091	183	79.57
GDFY	1.2031±0.2434	0.1412±0.1393	0.2408±0.2009	185	80.43
YX	1.2452±0.2504	0.1695±0.1393	0.2852±0.1967	200	86.96
YNMD	1.2315±0.2551	0.1589±0.1437	0.2665±0.2059	188	81.74
DZ	1.1233±0.1864	0.0911±0.1151	0.1625±0.1781	143	62.17
YL	1.1451±0.1839	0.1092±0.1120	0.1974±0.1693	179	77.83
Total	1.2311±0.2253	0.1649±0.1274	0.2850±0.1747	230	100

Ne = Effective number of alleles; He = Nei’s gene diversity; I = Shannon’s Information index;
Npl = The number of polymorphic loci; Ppl = The percentage of polymorphic loci.
