# Supplementary material for: Insight into the Migration Routes of Plutella xylostella in China Using mtCOI and ISSR Markers
Source: PLoS One. 2015 Jun 22;10(6):e0130905. doi: 10.1371/journal.pone.0130905 (PMC4476569; doi:10.1371/journal.pone.0130905)
Supplement: S4 Table — (PDF) [file pone.0130905.s005.pdf]

S4 Table Estimated gene diversity for 23 *Plutella xylostella* populations as indicated by 11 ISSR makers.

Total number of geographic populations	Ht	Hs	Gst	Nm
23	0.1649±0.0162	0.1507±0.0126	0.0858	5.3277

Ht: Total genetic diversity for species; Hs: Genetic diversity within population;
Gst: coefficient of gene differentiation; Nm: Gene flow among populations.
