# Supplementary material for: Insight into the Migration Routes of Plutella xylostella in China Using mtCOI and ISSR Markers
Source: PLoS One. 2015 Jun 22;10(6):e0130905. doi: 10.1371/journal.pone.0130905 (PMC4476569; doi:10.1371/journal.pone.0130905)
Supplement: S1 Fig — (PDF) [file pone.0130905.s001.pdf]

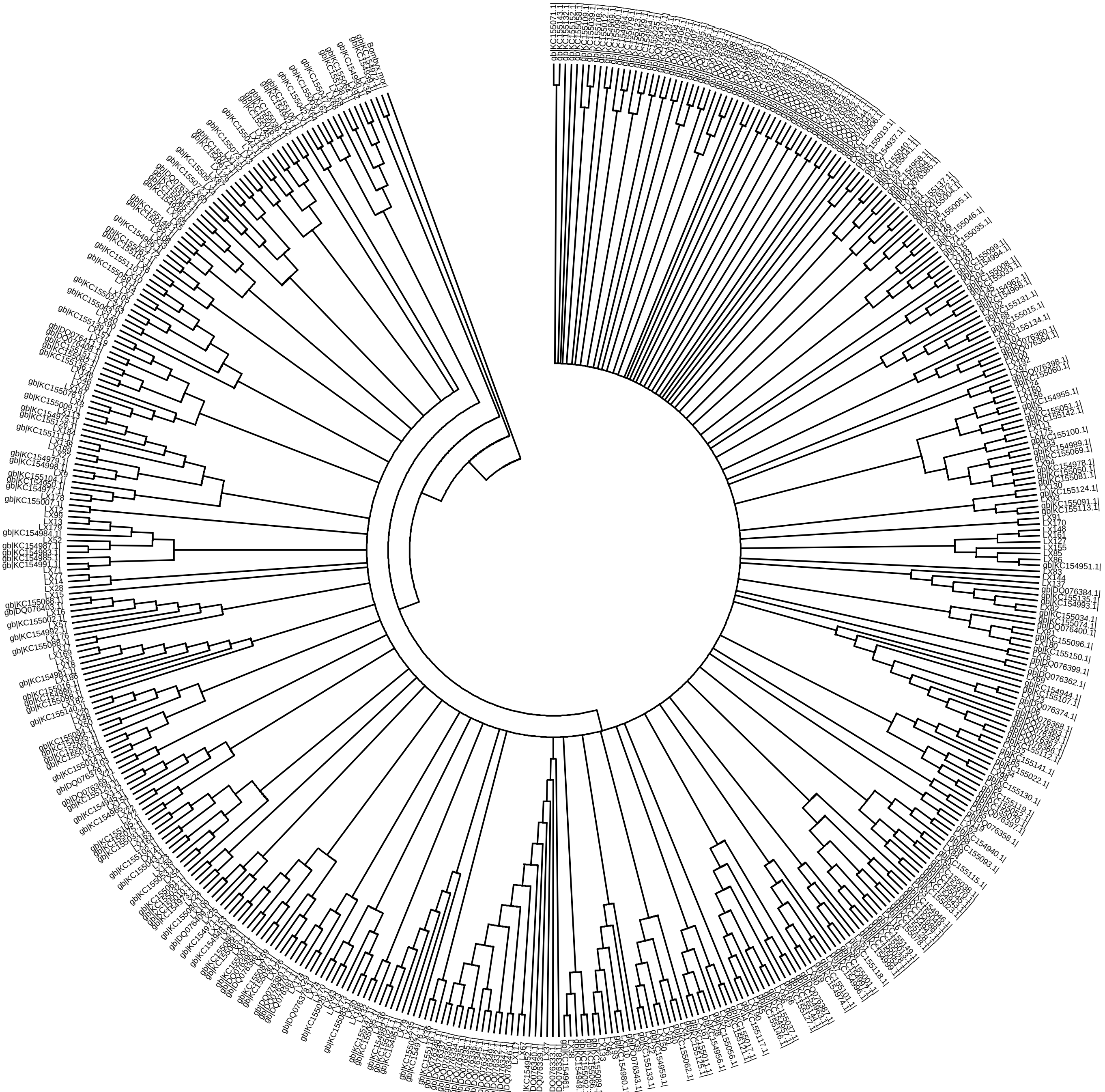

S1 Figure NJ tree of 324 mtCOI haplotypes of *Plutella xylostella* in China and South Korea.
